# Thorough evaluation for the new acute kidney injury criteria by Kidney Disease Improving Global Outcomes

**DOI:** 10.1186/cc12348

**Published:** 2013-03-19

**Authors:** J Izawa, S Uchino, T Fujii, T Arii, T Fukushima, S Kawano

**Affiliations:** 1 Jikei University School of Medicine, Tokyo, Japan

## Introduction

Two previous classifications of acute kidney injury (AKI) that are known as RIFLE criteria and AKIN criteria have shown that AKI is associated with increased morbidity and mortality. Differences in predicting ability for prognosis, however, have been reported. In 2012, Kidney Disease Improving Global Outcomes (KDIGO) created the new AKI criteria, combining RIFLE and AKIN criteria. However, such a combination might cause inconsistency among each definition in the criteria. We have investigated all of the definitions in the new KDIGO criteria in detail.

## Methods

This is a retrospective historical cohort study including adult patients admitted to the ICU (Jikei University, Tokyo, Japan) between January 2010 and October 2011. Patients undergoing chronic dialysis were excluded. KDIGO criteria were applied to all patients to diagnose AKI. Hospital mortality of patients with AKI diagnosed by the 10 definitions in the criteria was compared.

## Results

A total of 2,399 patients were evaluated. AKI occurred in 26.6% with standard definition of KDIGO; 18.0% with creatinine criteria alone; 15.5% with urine output alone. By multivariable analysis, each AKI stage was associated with hospital mortality: 5.6%, odds ratio 2.95, for Stage 1; 10.1%, odds ratio 5.52, for Stage 2; 30.2%, odds ratio 21.30, for Stage 3. Crude hospital mortality stratified by the 10 definitions showed increasing trends with stage progression. Mortality of the three definitions in Stage 1 was from 4.2% to 8.8%. Stage 2 had two definitions and their mortality was 12.1% and 12.9%. Stage 3 had five definitions and their mortality was from 20.5% to 55.6%.

## Conclusion

AKI defined by the new KDIGO criteria was associated with increased hospital mortality. In addition, definitions in the KDIGO criteria seem to be appropriate because of clear relations between mortality and stage progression.

